# Organization and characterization of a biosynthetic gene cluster for bafilomycin from *Streptomyces griseus* DSM 2608

**DOI:** 10.1186/2191-0855-3-24

**Published:** 2013-05-10

**Authors:** Jae Yoon Hwang, Hyo Sun Kim, Soo Hee Kim, Hye Ryeung Oh, Doo Hyun Nam

**Affiliations:** 1 College of Pharmacy, Yeungnam University, Dae-dong, Gyongsan 712-749, Korea

**Keywords:** *Streptomyces griseus*, Bafilomycin, Plecomacrolide antibiotic, Polyketide synthase

## Abstract

*Streptomyces griseus* DSM 2608 produces bafilomycin, an antifungal plecomacrolide antibiotic. We cloned and sequenced an 87.4-kb region, including a polyketide synthase (PKS) region, methoxymalonate genes, flavensomycinate genes, and other putative regulatory genes. The 58.5kb of PKS region consisting 12 PKS modules arranged in five different PKS genes, was assumed to be responsible for the biosynthesis of plecomacrolide backbone including 16-membered macrocyclic lactone. All the modules showed high similarities with typical type I PKS genes. However, the starting module of PKS gene was confirmed to be specific for isobutyrate by sequence comparison of an acyltransferase domain. In downstream of PKS region, the genes for methoxymalonate biosynthesis were located, among which a gene for FkbH-like protein was assumed to play an important role in the production of methoxymalonyl-CoA from glyceryl-CoA. Further the genes encoding flavensomycinyl-ACP biosynthesis for the post-PKS tailoring were also found in the upstream of PKS region. By gene disruption experiments of a dehydratase domain of module 12 and an FkbH-like protein, this gene cluster was confirmed to be involved in the biosynthesis of bafilomycin.

## Introduction

Plecomacrolide antibiotic is an unusual class of macrolide antibiotic which has a structural element of 6-membered hemiacetal ring connected to the macrolactone ring by C3-spacer (Dröse and Altendorf 1997). In early 1980s, two macrolides were discovered: one was bafilomycin showing antifungal activity (Werner et al. 1984) and the other concanamycin screened as an immunosuppressive compound (Kinashi et al. 1984). Later some other compounds in this category were further found from nature, including hygrolidin (Seto et al. 1984), setamycin (Otoguro et al. 1988), leucancidin (O’Shea et al. 1997), micromonospolide (Ohta et al. 2001), and so on.

In early days, plecomacrolide antibiotics were found to have a wide spectrum of activities including antifungal, antimalarial and antiparasitic activity as well as antibacterial activity against Gram-positive bacteria. Since Bowman et al. (1988) reported that bafilomycin shows a high-affinity inhibitory function on vacuolar-type ATPase (V-ATPase), this compound became a widely-used biochemical research tool to study the function of this type of ATPases. Interestingly, this class of antibiotics makes a clear distinctive inhibition of V-ATPase activity from other type of ATPases (P-ATPase and F-ATPase), which can promote the accumulation of autophagic vacuoles and trigger Bax-dependent autophagy (Shacka et al. 2006). This specific inhibitory function suggests the possibility of clinical application in the treatment of osteoporosis associated with excessive bone resorption (Farina and Gagliardi 2002;Xu et al. 2007).

The unusual structure of plecomacrolide antibiotics evokes researcher’s interests about their biosynthetic pathway. Schumann and his collegues (2004; 2007) reported the biosynthetic origin of bafilomycin and concanamycin by feeding experiments with ^13^C-labelled precursors. They assumed that the main backbone of plecomacrolide including macrolactone and hemiacetal ring is assembled from an isobutyrate starter unit, 7 propionate extender units, 2 acetate extender units, and 2 methoxyl C_2_ extender units, on the modular type I polyketide synthases (PKSs). They proposed that the methoxymalonyl CoA for methoxyl C_2_ extender units could be derived from glycerol through D-1,3-bisphosphoglycerate.

The first plecomacrolide antibiotic of which the biosynthetic gene cluster was reported is concanamycin A of *Streptomyces neyagawaensis* (Haydock et al. 2005). In this cluster, genes for a set of polyketide synthases encoding 14 modules and for deoxysugar biosynthesis were found. Recently, the genome sequence of *Kitasatospora setae* producing bafilomycin has been revealed (Ichikawa et al. 2010). Among 24 gene clusters for secondary metabolite biosynthesis, the predictive gene cluster for bafilomycin biosynthesis was located in the left subtelomeric region. However, the authors did not further characterize this gene cluster beyond indentifying its putative gene sequences.

Here we report the full gene cluster for bafilomycin biosynthesis from *Streptomyces griseus* DSM 2608, which was formerly reported as Tü 1922 (Werner et al. 1984;Hagenmaier et al. 1985). The involvement of this gene cluster in bafilomycin biosynthesis was also confirmed by gene disruption experiments.

## Materials and methods

### Strains, vectors, culture media and reagents

*Escherichia coli* XL1-blue, *E. coli* XL1-blue MRF’ and *E. coli* JM109 grown on Luria-Bertani (LB) medium at 37°C were used routinely for gene manipulation in this study. The cloning vectors pGEM-T-easy, pUC18 or pUC19 plasmid were generally employed for subcloning. For blue/white selection, 0.1 mM of isopropyl-D-thiogalactopyranoside (IPTG), and 80 μg ml^-1^ of 5-bromo-4-chloro-3-indolyl-β-D-galactopyranoside (X-Gal) were added on the medium, and for positive selection, appropriated antibiotic solutions were also supplemented. In gene disruption experiment, *E. coli* BW25113/pIJ790 cultured on SOC medium (2% trypton, 0.5% yeast extract, 0.05% NaCl, 2.5 mM KCl, 10 mM MgCl_2_, 20 mM glucose, pH 7.0) was used for homologous double-crossover recombination (Datsenko and Wanner 2000) and *E. coli* ET12567/pUZ8002 grown on LB medium for conjugation (Biermann et al. 1992). The induction of an *ara* promoter in pIJ790 plasmid was achieved by the addition of 10 mM arabinose to LB agar medium, and pIJ773 plasmid was used as a template for the construction of disruption cassette. The bafilomycin-producer *S. griseus* DSM 2608 was cultivated on tryptic soy broth (TSB) at 28°C for 3 days for simple culture, or mannitol soy flour (MS) agar (2% mannitol, 2% soy flour, 2% agar) for the selection of gene disruptants. The fermentation of this strain was carried out on the production agar medium (2% glucose, 1% soluble starch, 0.1% meat extract, 0.4% yeast extract, 2.5% soybean meal, 0.2% NaCl, 0.005% K_2_HPO_4_, pH 7.3) (Moon et al. 2003). The antifungal activity was measured using *Rhizoctonia solani* AG-1 (KACC 40401) grown on potato dextrose agar medium. The authentic bafilomycin A_1_ was purchased from Fermentek (Israel) and bafilomymycin B_1_ from Santa Cruz Biotech (USA).

### Preparation of probes for gene screening

For screening the bafilomycin biosynthetic gene cluster, three different gene fragments of ketosynthase (KS), aminolevulinate synthase (ALS) and FkbH-like protein were amplified from *S. griseus* chromosomal DNA based on their conserved domains. The chromosomal DNA of *S. griseus* DSM 2608 was isolated from mycelia following the procedure using 10% cetyltrimethylammonium bromide (CTAB) (Kieser et al. 2000). The primers used for gene amplification by polymerase chain reaction (PCR) were as follows : KS-F (5^′^-SBVBTTCGACGCSBSSTTCTTCG-3^′^), KS-R (5^′^-RCSAGSGASGASGAGCASGCSGTGTC-3^′^), ALS-F (5^′^-AGGAACTAGCGGATCTGCAC-3^′^), ALS-R1 (5^′^-ACGAAGATCGA GACGATGTG-3^′^), FkbH-F (5^′^-AARTGYCTSGTCTGGGACCTSGACRACACMCTSTGGC-3^′^), and FkbH-R (5^′^-GCGTTCATCTGGCTGGTGCGCAGGGTSAGTTCCTCGACCC-3^′^). The extension of the biosynthetic gene cluster, a new primer set was also designed as follows: ACL-F (5^′^-GAGGGGGAAGGCGTCCACGAACTCCACCCGGCGCGGGTACTTGTA-3^′^) and ALS-R2 (5^′^-AATTTTCCGGCGAGCCGGCCCAGTTCGAGGAACTCCCGTTTCGCAT-3^′^). A typical amplification conditions were as follows: an initial hot start at 94°C for 5 min; 35 cycles of amplification with each repeat of denaturation at 30 sec at 94°C, annealing at 52.5°C for 30 sec, and elongation at 72°C for 30 sec; and final completion at 72°C for 5 min. The amplified probes were radiolabeled with α-[^32^P]-dCTP (10 mCi ml^-1^; Perkin Elmer, USA) by DecaLabel™ DNA labeling kit (Thermo Scientific, UK), and purified by passing through a Elutip-D column (Schleicher & Shuell, Germany).

### Construction and screening of cosmid library of genomic DNA

The obtained chromosomal DNA was partially digested with *Mbo*I, and the DNA fragments larger than 23 kb were excised from agarose gel and then ligated with *Bam*HI-digested and dephosphorylated SuperCos1 cosmid vector. The ligated DNA products were then *in vitro* packaged using Gigapack III packaging extract (Stratagene, USA). After adding SM buffer (phage dilution buffer), the ligated cosmid DNAs (ca 5 μg) were then transfected into *E. coli* XL1-blue MRF’ grown overnight on LB broth supplemented with 10 mM MgSO_4_. The host cells were then incubated on LB liquid media containing ampicillin. The appeared colonies were transferred to Nylon membrane by placing it on the agar plate, and subjected to *in situ* hybridization with ^32^P-labeled probes (Sambrook et al. 1989).

### DNA sequencing and analysis

The selected recombinant cosmids were sent to Genotech Co., Inc. (Daejeon, Korea) for sequencing. The location of the open reading frames (ORFs) in the sequences was determined using FramePlot version 2.3.2 (http://www0.nih.go.jp/~jun/cgi-bin/frameplot.pl) (Ishikawa and Hotta 1999). The homology search with the obtained DNA or protein sequences was performed with BLAST program (http://blast.ncbi.nlm.nih.gov). The multiple alignments of DNA or protein sequences were performed with EBI–ClustalW2 program (http://www.ebi.ac.uk/Tools/clustalw2/index.html).

### Gene disruption

The gene disruption was performed following the protocol of John Innes Centre (Gust et al. 2003). Employing pIJ773 plasmid as a template, the disruption cassette for a dehydratase domain of module 12 was amplified using forward primer (5^′^-GCCTGCGGGTCGACTGGGAGCGGCTGTTCGCGGGGACCATTCCGGGGATCCGTCGACC-3^′^) and reverse primer (5^′^-GGTGTTCGAGCTGGGCCGCGGCGAGCATGCCCCGAACGGTGTAGGCTGGAGCTGCTTC-3^′^), and that for a FkbH-like protein was also amplified using forward primer (5^′^-AGCGGGCCCGCGTCCGACGAGACGAGGAAGGACGACATGATTCCGGGGATCCGTCGACC-3^′^) and reverse primer (5^′^-CGGGCCGGAGCCCGGGAGCACCGGCCGCCGCGGCGGTCATGTAGGCTGGAGCTGCTTC-3^′^). These extended apramycin disruption cassettes were introduced by electroporation into cell suspension of *E. coli* BW25113/pIJ790 harboring pSGB20 cosmid, which were grown in the presence of arabinose. After incubation overnight at 37°C, a single clone was selected in which the target gene was replaced by the disruption cassette. For conjugal transfer to bafilomycin-producing *S. griseus,* the PCR-targeted disruption cosmid was purified and transformed in *E. coli* ET12567/pUZ8002 by electroporation, and then transferred by intergeneric conjugation to wild strain. The conjugation was tried using *Streptomyces* spores obtained from MS agar plate and then heated at 50°C for 10 min to induce germination. The mixture of *E. coli* ET12567/pUZ8002 cells and heated spores were spread on MS agar plate supplemented with 10 mM MgCl_2_ and apramycin at 30°C overnight. One day after, the plates were overlaid with 300 μL of apramycin and nalidixic acid and incubated for 5 days more. For selection of the disrupted colonies, replica-plates with single colonies were made onto a nutrient agar containing nalidixic acid and apramycin for positive selection, and the other onto a nutrient agar containing nalidixic acid, apramycin and kanamycin for negative selection.

### Metabolite analysis

The wild strain and the gene disuptants were cultured on the production agar plates (pH 7.3) at 28°C for 7 days. The plates were minced and directly extracted twice with ethyl acetate. After drying on anhydrous sodium sulfate and concentration *in vacuo*, the extracts dissolved in chloroform-methanol (9:1) were chromatographed on Kieselgel 60 (0.063~0.200 mm, Merck, Germany) with the same solvent. The fractions of wild-type showing the antimicrobial activity against *R. solani* were collected. Once more purification was performed by chromatography on Lichroprep Si 60 (40–63 μm; Merck, Germany) with the solvent system of chloroform-methanol (95:5), and the antimicrobial fractions were also collected. In case of disruptants, the fractions corresponding to the same elution number were collected. The fractions were dried and applied on high-performance liquid chromatography (SCL-10A_VP_ system, Shimadzu Co., Japan) using YMC-Pack Pro C_18_ reverse-phase column (250 mm × 4.6 mm l.D., S–5 μm, 12 nm) (YMC Co., Ltd., Japan). The isocratic mobile phase of acetonitrile-methanol (9:1) was pumped at a flow rate of 1 ml min^-1^, and the eluent was detected at 254 nm.

## Results

### Screening of a cosmid library of *S. griseus* genomic DNA

As screening probes, the DNA fragments of KS, ALS and FkbH-like protein were firstly amplified from *S. griseus* DSM 2608 chromosomal DNA. The gene amplification yielded DNA fragments having about 590 bp for KS gene, 520 bp for ALS gene and 600 bp for FkbH-like protein gene, respectively (Additional file 1: Figure S1). The amplified KS probe had a highly similar nucleotide sequence over 95% with *TrdAIII* gene of *Streptomyces* sp. SCSIO1666 (GenBank No. HQ852227.1) and *TamAIII* gene of *Streptomyces* sp. 307–9 (GenBank No. GU385216.1). The amplified ALS probe also showed the high homology around 92% in nucleotide sequences with *bfmK* gene of *Kitasatospora setae* KM-6054 (GenBank No. NC_016109.1) and *bafZ* gene of *S. lohii* (GenBank No. GU390405.1). The amplified probe for FkbH-like protein fragment also exhibited the high homology over 90% in nucleotide sequences with *bfmE* gene of *K. setae* KM-6054 (GenBank No. NC_016109.1) and *bafE* gene of *S. lohii* (GenBank No. GU390405.1).

The cosmid library was made in SuperCos1 vector at *Bam*HI site by ligating the large fragments of chromosomal DNA partially digested with *Mbo*I. The recombinant cosmid were *in vitro* packaged and transfected into *E. coli* XL1-blue MRF' strain. Around 5×10^4^ to 5×10^5^ transfected bacterial colonies per μg DNA were counted on LB plate containing ampicillin. The obtained cosmid clones were diluted 10^4^-10^6^ fold to give around 5×10^3^ colonies per LB plate, and screened the bafilomycin biosynthetic gene cluster using the amplified probes labeled with α-[^32^P]- dCTP.

One positive clone, pSGB1 was firstly screened based on the multiple strong signals with KS probes, which indicates the presence of type I multi-modular PKS responsible for the biosynthesis of type I polyketide molecule (Additional file 1: Figure S2). To extend the biosynthetic gene cluster in pSGB1 cosmid by chromosome walking, the amplified DNA fragments of ALS gene and FkbH-like protein gene were employed as the second probes for colony hybridization. Among 63 positive cosmid clones screened using KS probes, only 2 cosmid clones were hybridized with ALS probe and only 3 clones with FkbH probe during the second screening. As shown in Additional file 1: Figure S2, the chosen cosmid clones, pSGB23 and pSGB20, gave a strong signal with ALS probe or FkbH probe in addition to KS probe.

### Gene organization of bafilomycin biosynthetic gene cluster

**Figure 1:**
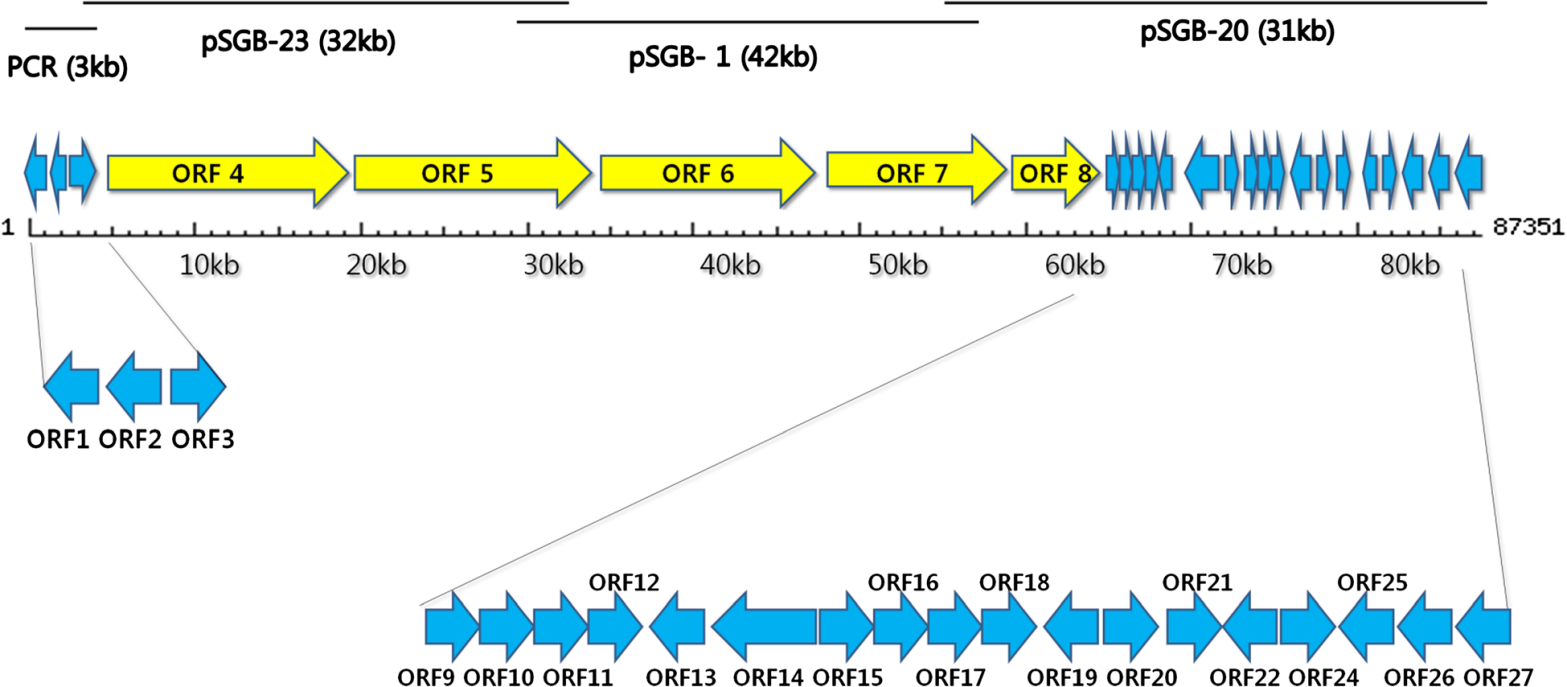
**Organization of bafilomycin biosynthetic gene cluster.** The overall gene cluster was made by assembly of pSGB-1, pSGB-20 and pSGB-23 cosmids. The upstream region was further amplified by using ACL and ALA primers. The total length of the cloned gene cluster was 87,324 bp, which was deposited in GenBank under accession number KC514471. In this gene cluster, 27 ORFs including 5 modular polyketide synthase genes were found, as described in Table 1.

By overlapping and combining the nucleotide sequences of pSGB-1 (42 kb), pSGB-20 (34 kb), and pSGB-23 cosmid clones (31 kb), a contiguous 87.4 kb genetic locus encoding 27 ORFs was drawn up as a complete gene cluster for bafilomycin biosynthesis (Figure 1). The putative functions of 27 ORFs present in this contiguous sequence were analyzed by BLAST search (Table 1). The gene sequence was submitted as GenBank accession number KC514471.

**Table 1:** **Gene organization of bafilomycin biosynthetic gene cluster of *Streptomyces griseus *DSM 2608**

**ORF**	**Gene product**	**Number of amino acid**	**Deduced function**	**The closest gene**	**Gene strain**	**Homology (%)**
				**(GenBank number)**		
ORF1	BafBI	460	acyl-CoA ligase (partial)	ADC79613.1	*Streptomyces lohii*	93
ORF2	BafBII	513	amide synthetase	ADC79614.1	*Streptomyces lohii*	96
ORF3	BafBIII	415	5-aminolevulinate synthase	ADC79615.1	*Streptomyces lohii*	98
ORF4	BafSI	5016	modular polyketide synthase I	ADC79616.1	*Streptomyces lohii*	93
ORF5	BafSII	5089	modular polyketide synthase II	YP_004909056.1	*Kitasatospora setae*	85
ORF6	BafSIII	3981	modular polyketide synthase III	ADC79618.1	*Streptomyces lohii*	94
ORF7	BafSIV	3627	modular polyketide synthase IV	ADC79619.1	*Streptomyces lohii*	89
ORF8	BafSV	2106	modular polyketide synthase V	ADC79620.1	*Streptomyces lohii*	93
ORF9	BafAI	297	3-hydroxyacyl-CoA dehydrogenase (NAD-dependent)	ADC79621.1	*Streptomyces lohii*	97
ORF10	BafAII	94	acyl carrier protein	ADC79622.1	*Streptomyces lohii*	97
ORF11	BafAIII	364	acyl-CoA dehydrogenases (FAD-dependent)	ADC79623.1	*Streptomyces lohii*	98
ORF12	BafAIV	367	FkbH-like protein	ADC79624.1	*Streptomyces lohii*	97
ORF13	BafAV	221	O-methyltransferase	ADC79625.1	*Streptomyces lohii*	95
ORF14	BafRI	610	AfsR family transcriptional regulator	ADC79626.1	*Streptomyces lohii*	96
ORF15	BafT	254	Thioesterase II	ADC79627.1	*Streptomyces lohii*	95
ORF16	BafRII	118	LuxR-like protein	ADC79628.1	*Streptomyces lohii*	91
ORF17	BafCII	321	malonyl transferase	ADC79629.1	*Streptomyces lohii*	97
ORF18	BafCI	360	Putative CoA ligase	ADC79630.1	*Streptomyces lohii*	97
ORF19		511	XRE family transcriptional regulator	ZP_04709597.1	*Streptomyces roseosporus*	98
ORF20		506	2-methylcitrate reductase	ZP_04709598.1	*Streptomyces roseosporus*	95
ORF21		379	methylcitrate synthase	ZP_04709599.1	*Streptomyces roseosporus*	94
ORF22		224	GntR family transcriptional regulator	ZP_11438254.1	*Mycobacterium abscessus*	53
ORF23		285	methylcitrate lyase	ZP_04709600.1	*Streptomyces roseosporus*	91
ORF24		491	putative transcriptional regulator	ZP_19189491.1	*Streptomyces ipomoeae*	71
ORF25		846	Osmosensitive K+ channel histidine kinase	ZP_04995839.1	*Streptomyces* sp. Mg1	84
ORF26		225	K^+^-transporting ATPase subunit C	ZP_04710866.1	*Streptomyces roseosporus*	82
ORF27		486	K^+^-transporting ATPase subunit B (partial)	YP_001824190.1	*Streptomyces griseus subsp. griseus*	91

Five genes for BafSI to BafSV were deduced to be the type Ι PKS genes responsible for the biosynthesis of main macrolactone backbone of bafilomycin. At the upstream of gene cluster, three genes for BafBI to BafBIII were presumed to be engaged in the biosynthesis of flavensomycinyl moiety of bafilomycin B_1_. At the downstream of PKS genes, the genes involved in the biosynthesis of methoxymalonyl-CoA from glycerol as a polyketide precursor (Chan and Thomas 2010) were also found from BafAI to BafAV. Two genes for transcription regulators in the families of SARP and LuxR were also found at the downstream of gene cluster. However, it is uncertain whether ten gene products located at downstream of gene cluster are related to bafilomycin biosynthesis or not.

### Functional characterization of bafilomycin biosynthetic gene cluster

In order to confirm whether the cloned gene cluster is really involved in the biosynthesis of bafilomycin, the gene for a dehydratase domain of module 12 (DH12) of PKS and an FkbH-like gene in *S. griseus* chromosome was disrupted by PCR-targeted gene disruption method (Gust et al. 2003). Firstly the disruption cassettes containing 5^′^-flanking upstream region and 3′-flanking downstream region for gene disruption, two FRT sites, apramycin resistance gene for selection, and *ori*T for conjugation in the size of 1.4 kb were amplified using pIJ773 plasmid as a template (Additional file 1: Figure S3). Those amplified disruption cassettes were used for disruption of each gene in pSGB20 cosmid by homologous recombination in *E. coli* BW25113/pIJ790. The disrupted cosmid, pSGB20-DH12-apr or pSGB20-FkbH-apr, was transformed again into *E. coli* ET12567 containing conjugal plasmid pUZ 8002. This *E. coli* strain was conjugated in the presence of MgCl_2_ with wild-type *S. griseus* spores. After 5 day-cultivation, the positive conjugants, *S. griseus* DH12-apr or *S. griseus* FkbH-apr, which was resistant to apramycin but sensitive to kanamycin were selected. The chromosomal DNAs of disrupted strains were confirmed by PCR analysis (Additional file 1: Figure S4).

**Figure 2:**
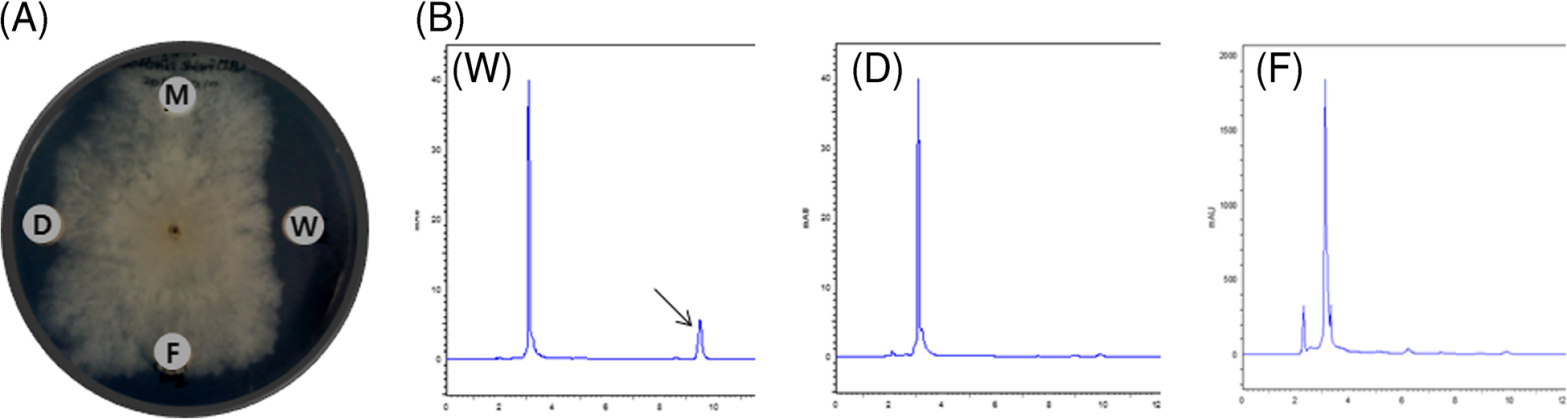
**Metabolte analysis of wild strain and gene disruptants.** (**A**) Antifungal activity of metabolites against *Rhizoctonia solani* AG-1 (KACC 40111). M, methanol (control); W, wild strain *S. griseus* DSM 2608; D, gene disruptant of DH domain in PKS module 12; F, FkbH gene disruptant. (**B**) HPLC pattern of metabolites. The antifungal fractions were eluted by acetonitrile-methanol (9:1) through C_18_ reverse-phase column. The retention time of bafilomycin B_1_ was 9.85 min.

After cultivation of wild strain and two disruptants, the antimicrobial activity on *R. solani* was examined. Even though the culture broth of wild strain showed the growth inhibitory activity, those of two deleted mutants, DH12-apr and FkbH-apr did not (Figure 2A). The culture broths were extracted with ethyl acetate and the metabolites were purified sequentially through two different silica gel column chromatography. The HPLC analysis of extract from wild strain showed the typical peak for bafilomycin B_1_ but no peak for bafilomycin A_1_. However in the extracts of two disruptants, neither bafilomycin A_1_ nor bafilomycin B_1_ was found in HPLC profiles (Figure 2B). The above results imply that the cloned gene cluster really commands the bafilomycin biosynthesis in *S. griseus* DSM 2608.

## Discussion

Plecomacrolide antibiotic is an unusual macrolide antibiotic which has a structural element of 6-membered hemiacetal ring connected to the macrolactone ring. The unusual structure drew the scientist’s interests about their biosynthetic pathway. Among plecomacrolide antibiotics, the concanamycin biosynthetic gene cluster was firstly reported from *S. neyagawaensis* (Haydock et al. 2005), and the biosynthetic pathway of plecomacrolide backbone was partly deduced.

We cloned an 87.4 kb chromosomal region of *S. griseus* DSM 2608, including presumable biosynthetic gene cluster of bafilomycin. This gene cluster was confirmed by metabolite analysis after gene disruption.

**Figure 3:**
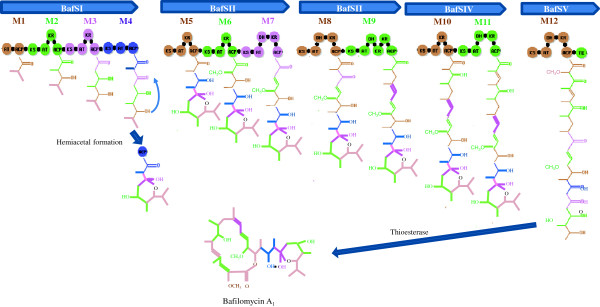
**Architecture of modular polyketide synthase in the biosynthesis of bafilomycin A**_**1**_**.** Five type Ι PKS genes encodes totally 12 modules responsible for bafilomycin biosynthesis. The first module of BafSI starts the polyketide synthesis from isobutyl group, and extends its length by incorporating 1 acetyl group and 2 propionyl groups, making 9 carbon backbone. The hemiacetal ring can be earlier formed, and further polyketide extension continues until biosynthesis is terminated by thioesterase of the last module of BafSV.

Among five type Ι PKS genes ranged in 58.5kb, the first type Ι PKS gene *bafSI* was comprised of 4 PKS modules, the second *bafSII* of 3 PKS modules, the third *bafSIII* of 2 PKS modules, the fourth *bafSIV* of 2 PKS modules, and the fifth *bafSV* of 1 PKS module having thioesterase domain (Figure 3). Thus one starter unit and eleven extender modules are involved in the biosynthesis of bafilomycin main backbone.

**Figure 4:**
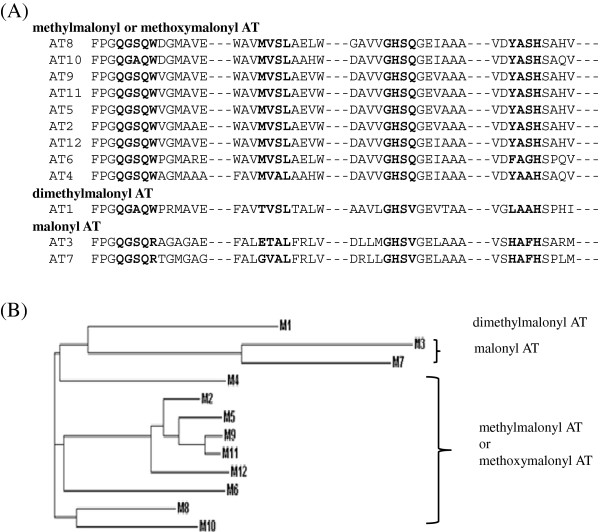
**Comparison of nucleotide sequences at the active sites in each acyltransferase (AT) domain.** (**A**) Sequence alignment of each domain by ClustalW. (**B**) Phylogenic dendrogram of each domain. The first AT domain in module 1 (AT1) was highly similar with AT domains specific for dimethylmalonyl-CoA to incorporate isobutyl group. Two AT domains of module 3 (AT3) and module 7 (AT7) was highly homologous with other AT domains specific for malonyl-CoA to incorporate acetyl group. The other nine AT domains exhibited the similar sequences with other AT domains specific for methylmalonyl-CoA to incorporate propionyl group. Contrastly, AT domains of module 6 (AT6) and module 12 (AT12) shows high similarity with AT domains specific for methylmalonyl-CoA, but they were assumed to be specific for methoxymalonyl-CoA to incorporate methoxyacetyl group, based on the chemical structure of bafilomycin.

The substrate specificity of an acyltransferase (AT) domain of each module determines the incorporation of specific acyl-CoA precursors. In comparison of active sites of AT domains in this gene cluster showed that AT domain of the starter module 1 has the conserved sequences specific for dimethylmalonyl-CoA to incorporate isobutyl group (Figure 4). In contrast, AT domains of module 3 and module 7 has the high homology with other AT domains specific for malonyl-CoA to incorporate acetyl group. The other nine AT domains in the bafilomycin PKS genes exhibited the similar sequences with other AT domains specific for methylmalonyl-CoA to incorporate propionyl group. From chemical structure of bafilomycin, it can be deduced that AT domains of module 6 and module 12 might be specific for methoxymalonyl-CoA to incorporate methoxyacetyl group. However, any significant sequence differences between AT domains specific for methoxymalonyl-CoA and those for methylmalonyl-CoA was not found.

Thus the first PKS having 4 modules encoded from *bafSI* starts the polyketide synthesis from isobutyl group, and extend its length by incorporating 1 acetyl group and 2 propionyl groups, making 9 carbon backbone. Considering the chemical structure of bafilomycin, the hemiacetal ring can be formed at this step before further polyketide extension.

The polyketide biosynthesis is continuously mediated by following PKS proteins directed by BafSII, BafSIII, BafSIV, and the final BafSV. Those PKSs are comprised of 8 modules which can incorporate 1 acetyl group, 2-methoxyacetyl groups and 5 propionyl groups during polyketide extension. Since the incorporation of the methoxy groups at C-2 and C-14 position of bafilomycin macrolactone is originated from glycerol by feeding experiments of radiolabeled precursors (Schuhmann and Grond 2004;Schuhmann et al. 2007), it is imagined that module 6 and module 12 might transfer 2-methoxyacetyl group by methoxymalonyl-CoA originated from glycerol.

**Figure 5:**
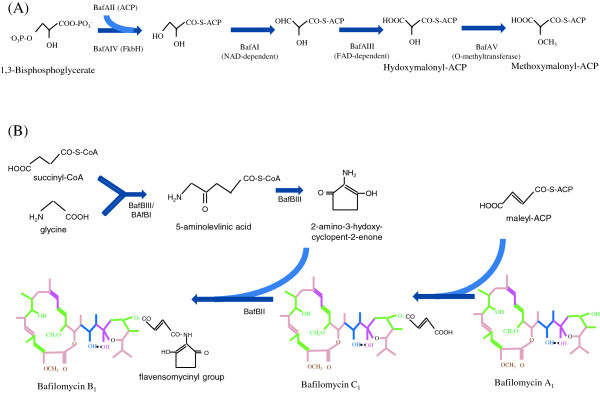
**The presumable post-PKS tailoring pathway of bafilomycin B**_**1**_**.** (**A**) The presumable biosynthetic pathway of methoxymalonyl-ACP from 1,3-bisphophoglycerate. (**B**) The presumable biosynthetic pathway of flavensomycinyl group from glycine, succinyl-CoA and fumaryl-CoA.

The generation of methoxymalonyl-CoA from 1,3-bisphosphoglycerate was already proven in the biosynthesis of ansamitocin (Wenzel et al. 2006), zwittermicin (Chan and Thomas 2010) and tetronomycin (Sun et al. 2008). The fed glycerol is firstly converted into 1,3-bisphosphoglycerate for primary metabolism, which can be acylated on acyl carrier protein (ACP) to produce glyceryl-ACP. This glyceryl-ACP is further oxidized to hydroxymalonyl-ACP by NAD-dependent acyl-CoA dehydrogenases and FAD-dependent acyl-CoA dehydrogenase. Finally methoxymalonyl-ACP is produced by methylation of hydroxymalonyl-ACP by O-methyltransferase (Figure 5A).

The compound made after macrolactone formation is bafilomycin A_1_, which can be modified to bafilomycin C_1_ by esterification with fumaryl group at hydroxyl group in hemiacetal ring, and further to bafilomycin B_1_ by linking 2-amino-3-hydroxy-cyclopenta-2-enone, finally building-up flavensomycinyl moiety (Figure 5B). In the bafilomycin biosynthetic gene cluster cloned here, the genes responsible for the biosynthesis of 2-amino-3-hydroxy-cyclopenta-2-enone were also found. The 2-amino-3-hydroxy-cyclopenta-2-enone production through 2 steps of enzyme reaction was reported previously (Zhang et al. 2010). Firstly, 5-aminolevulinate synthase can catalyze the formation of 5-aminolevulinic acid from glycine and succinyl-CoA, which is then converted to 5-aminolevulinyl-CoA by acyl-CoA ligase. Even though 5-aminolevulinyl-CoA is spontaneous converted to 2,5-piperidinedione due to its instability, it is more easily cyclized to 2-amino-3-hydroxy-cyclopenta-2-enone in the presence of 5-aminolevulinate synthase. Finally this compound can be connected with fumaryl group by amide synthetase.

For the incorporation of fumaryl group into flavensomycinyl moiety, it was assumed that fumaric acid is firstly converted to fumaryl-CoA by BafCI (acyl CoA ligase), and then connected to bafilomycin polyketde backbone by BafCII (malonyl transferase). However, the catalytic properties of those enzymes should be further characterized.

## Competing interests

The authors declare that they have no competing interests.

## Supplementary Material

Additional file 1: Figure S1 The amplified products of KS, ALA, and FkbH probes. Lane M, 1 kb DNA ladder; lane 1, the amplified KS probe (590 bp); lane 2, the amplified FkbH probe (600 bp); lane 3, the amplified ALS probe (520 bp). **Figure S2.** Screening of bafilomycin biosynthetic gene cluster. pSGB1 cosmid clone was selected based on the multiple bands binding with KS probe. pSGB20 cosmid clone was isolated by using KS probe and FkbH probe, and pSGB23 cosmid clone was screened by using KS probe and ALS probe. (A) agarose gel electrophoresis pattern; (B) Southern blotting result. Left lane, 1 kb DNA ladder; right lane, each cosmid DNA digested with *Nco*I. **Figure S3.** The amplified disruption cassettes for gene inactivation for DH domain in PKS module 12 and FkbH gene. Lane M, 1 kb DNA ladder; lane 1, the amplified disruption cassette for DH domain of PKS module 12 (1.3 kb); lane 2, the amplified disruption cassette for FkbH gene (1.3 kb). **Figure S4.** Confirmation of disrupted mutants by gene amplification from chromosomal DNA. (A) Confirmation of DH domain of PKS module 12. Lane M, 100 bp DNA ladder; lane 1, the amplified gene from pIJ773 plasmid (template for disruption cassette); lane 2, the amplified DNA from the wild strain chromosome (1661 bp); lane 3, the amplified DNA from the disrupted mutant chromosome (1652 bp). (B) Confirmation of FkbH gene. Lane M, 100 bp DNA ladder; lane 1, the amplified DNA from pIJ773 plasmid (template for disruption casstte); lane 2, the amplified DNA from the wild strain chromosome (1280 bp); lane 3, the amplified DNA from the disrupted mutant chromosome (1554 bp). Click here for file
