# Supplementary material for: Organization and characterization of a biosynthetic gene cluster for bafilomycin from Streptomyces griseus DSM 2608
Source: AMB Express. 2013 May 10;3:24. doi: 10.1186/2191-0855-3-24 (PMC3679948; doi:10.1186/2191-0855-3-24)
Supplement: Additional file 1: Figure S1 — The amplified products of KS, ALA, and FkbH probes. Lane M, 1 kb DNA ladder; lane 1, the amplified KS probe (590 bp); lane 2, the amplified FkbH probe (600 bp); lane 3, the amplified ALS probe (520 bp). Figure S2. Screening of bafilomycin biosynthetic gene cluster. pSGB1 cosmid clone was selected based on the multiple bands binding with KS probe. pSGB20 cosmid clone was isolated by using KS probe and FkbH probe, and pSGB23 cosmid clone was screened by using KS probe and ALS probe. (A) agarose gel electrophoresis pattern; (B) Southern blotting result. Left lane, 1 kb DNA ladder; right lane, each cosmid DNA digested with NcoI. Figure S3. The amplified disruption cassettes for gene inactivation for DH domain in PKS module 12 and FkbH gene. Lane M, 1 kb DNA ladder; lane 1, the amplified disruption cassette for DH domain of PKS module 12 (1.3 kb); lane 2, the amplified disruption cassette for FkbH gene (1.3 kb). Figure S4. Confirmation of disrupted mutants by gene amplification from chromosomal DNA. (A) Confirmation of DH domain of PKS module 12. Lane M, 100 bp DNA ladder; lane 1, the amplified gene from pIJ773 plasmid (template for disruption cassette); lane 2, the amplified DNA from the wild strain chromosome (1661 bp); lane 3, the amplified DNA from the disrupted mutant chromosome (1652 bp). (B) Confirmation of FkbH gene. Lane M, 100 bp DNA ladder; lane 1, the amplified DNA from pIJ773 plasmid (template for disruption casstte); lane 2, the amplified DNA from the wild strain chromosome (1280 bp); lane 3, the amplified DNA from the disrupted mutant chromosome (1554 bp). [file 2191-0855-3-24-S1.docx]

**Legend of Supplementary Figures**

**Fig. S1.** The amplified products of KS, ALA, and FkbH probes. Lane M, 1 kb DNA ladder; lane 1, the amplified KS probe (590 bp); lane 2, the amplified FkbH probe (600 bp); lane 3, the amplified ALS probe (520 bp).

**Fig. S2.** Screening of bafilomycin biosynthetic gene cluster. pSGB-1 cosmid clone was selected based on the multiple bands binding with KS probe. pSGB-20 cosmid clone was isolated by using KS probe and FkbH probe, and pSGB-23 cosmid clone was screened by using KS probe and ALS probe. (A) agarose gel electrophoresis pattern; (B) Southern blotting result. Left lane, 1 kb DNA ladder; right lane, each cosmid DNA digested with *Nco*I.

**Fig. S3.** The amplified disruption cassettes for gene inactivation for DH domain in PKS module 12 and FkbH gene. Lane M, 1 kb DNA ladder; lane 1, the amplified disruption cassette for DH domain of PKS module 12 (1.3 kb); lane 2, the amplified disruption cassette for FkbH gene (1.3 kb).

**Fig. S4.** Confirmation of disrupted mutants by gene amplification from chromosomal DNA. (A) Confirmation of DH domain of PKS module 12. Lane M, 100 bp DNA ladder; lane 1, the amplified gene from pIJ773 plasmid (template for disruption cassette); lane 2, the amplified DNA from the wild strain chromosome (1661 bp); lane 3, the amplified DNA from the disrupted mutant chromosome (1652 bp). (B) Confirmation of FkbH gene. Lane M, 100 bp DNA ladder; lane 1, the amplified DNA from pIJ773 plasmid (template for disruption casstte); lane 2, the amplified DNA from the wild strain chromosome (1280 bp); lane 3, the amplified DNA from the disrupted mutant chromosome (1554 bp).

**Fig. S1**

**Fig. S2**

**Fig. S3**

**Fig. S4**
